# Association genetics and genomic prediction for resistance to root rot in a diverse collection of *Pisum sativum* L.

**DOI:** 10.1186/s12870-025-07803-0

**Published:** 2025-12-19

**Authors:** Daniel Ariza-Suarez, Lukas Wille, Pierre Hohmann, Valentin Gfeller, Michael Schneider, Matthew W. Horton, Monika M. Messmer, Bruno Studer

**Affiliations:** 1 Molecular Plant Breeding, Institute of Agricultural Sciences, ETH Zurich, Universitaetstrasse 2, 8092 Zurich, Switzerland; 2 Department of Crop Sciences, Research Institute of Organic Agriculture (FiBL), Ackerstrasse 113, 5070 Frick, Switzerland; 3 Department of Biology, Healthcare and the Environment, Faculty of Pharmacy and Food Sciences, Universitat de Barcelona, Barcelona, 08028 Spain

**Keywords:** Genome-wide association studies, Genomic prediction, Candidate resistance genes, Pea (*Pisum sativum* L.), Root rot

## Abstract

**Background:**

Root rot is one of the most threatening diseases to pea production. Root rot is caused by several interacting soil-borne pathogens which makes it challenging to manage. Breeding for resistance is a promising approach for sustainable pea production. While quantitative trait loci (QTL) for resistance against individual pathogens have been identified, the genetic basis underlying resistance against the pathogen complex is poorly understood.

**Results:**

Using a previously described diverse panel of 254 pea genotypes and 18k single nucleotide polymorphism (SNP) markers, we identified a novel QTL for resistance to root rot on chromosome chr6LG2. This QTL co-locates with a *mitochondrial Rho GTPase* and an *F-box* gene model, which are promising candidates for disease control. A whole-genome prediction model explained up to 53% of the phenotypic variation and reached predictive abilities of up to 0.51 for root rot-related traits. We found that plant height and shoot biomass were unreliable indicators of plant health. Instead, these traits were related to the Mendelian *Le* locus, which controls stem length.

**Conclusions:**

Our results provide new insights into the genetic basis of quantitative root rot resistance in pea and provide novel tools that could accelerate the development of resistant pea lines through marker-assisted and genomic selection.

**Supplementary Information:**

The online version contains supplementary material available at 10.1186/s12870-025-07803-0.

## Background

Pea (*Pisum sativum* L.) is an important pulse crop for human consumption. Globally, around 14,166 Mt of dry peas were produced in 2022, ranking third after common bean and chickpea production [1]. Pea production has also played a long-standing role in the European food system, but has declined since 1990. Given its desirable nutritional quality and ability to fix nitrogen, pea has the potential to play a central role in healthy and sustainable human diets, as well as reducing greenhouse gas emissions associated with mineral fertilizer use [2]. However, one of the most threatening factors for pea production are soil-borne diseases [3]. These include root rot caused by a complex of fungal and oomycete species such as *Aphanomyces euteiches*, *Fusarium solani*, *Fusarium avenaceum*, *Fusarium graminearum*, *Thielaviopsis* spp., *Rhizoctonia* spp., *Didymella* spp. and *Pythium* spp. [4–6], also referred to as the pea root rot complex. These pathogens are responsible for severe seed and root rot, damping-off and seedling blight, leading to strong yield reductions [7]. However, they are not the dominant taxa when analyzing the root microbiome composition [8, 9]. Management of root rot generally uses a combination of crop rotation and planting of certified and fungicide-treated seeds [10]. However, the disease remains a threat to pea production. In this sense, breeding for resistant genotypes is seen as a sustainable approach for controlling root rot in pea [11, 12].

Breeding for resistance to root rot is a complex task. The phenotypic expression of the disease and the inheritance of resistance exhibit a quantitative behavior [9, 13]. In addition, the root microbiome plays an important role in plant health by developing pathogenic, mutualistic or commensal interactions with the plant host [14]. However, partial resistance has been reported in some pea genotypes [3], and molecular tools have been useful in exploiting resistance QTL for plant breeding. For example, marker-assisted backcrossing resulted in near-isogenic lines carrying single or combined alleles from different resistance QTL to *A. euteiches* [15]. This is a significant advancement, as selecting for quantitative disease resistance is often related to durability but is difficult to achieve [16–18]. Moreover, the recent availability of high-quality reference genomes is facilitating the identification of favorable alleles underlying phenotypic variation and accelerating breeding against root rot through genomics-assisted selection [19, 20].

Several studies have reported the identification of multiple QTL for individual resistance to either *A. euteiches, F. solani*, *F. avenaceum* or *F. graminearum* [3, 21–24]. While these studies have been an important step towards implementing more efficient resistance breeding strategies, they have some limitations. First, these studies relied on screening assays in which a single or a few isolates of individual pathogens were inoculated onto sterile substrates. While those protocols favor the reproducibility of results, they often fail to reflect natural infestation in the soil, where complex interactions between the plant and the microbiome contribute to plant resistance [6]. Second, most of these QTL were identified in biparental mapping populations, where the allelic diversity in such diploid, autogamous species is limited. Further, biparental QTL studies often have a limited mapping resolution and require the introgression of the beneficial resistance allele into breeding-relevant germplasm. Finally, most of these studies were carried out in the absence of a high-quality reference genome. Therefore, efficient exploitation of these QTL to accelerate disease resistance breeding has been limited. In fact, the genes involved in resistance to root rot are largely unknown, and only recent studies have highlighted the identification of candidate genes [25]. The use of a globally diverse panel of genotypes grown under controlled conditions on naturally infested soils could provide new insights into the resistance mechanisms of root rot and present new robust candidate loci for use in marker-assisted and genomic selection.

The main goal of this study was to characterize the genetic basis of resistance to root rot in pea using a diverse collection previously described for quantitative resistance. To this end, we aimed to *i*) identify QTL and their underlying candidate genes associated with disease resistance using the recently available genomic resources; *ii*) compare different surrogate measures of plant health for association mapping and *iii*) explore the use of a whole-genome regression model to complement the association mapping approach and validate its use for genomic prediction purposes.

## Methods

### Plant material and phenotypic data

This study used the plant material and phenotypic results reported by Wille et al. [9]. Briefly, a panel of 261 pea genotypes was assembled and tested for resistance to root rot. The panel contained full-leaf and semi-leafless genotypes comprising 177 genebank accessions from the USDA-ARS GRIN Pea Core Collection, which were selected based on available characteristics like origin, morphological traits and agronomic potential. In addition, the panel included 47 advanced breeding lines from a private organic breeding organization (Getreidezüchtung Peter Kunz, Switzerland) and 37 registered cultivars (cv.) from Europe. These cultivars included the cv. ‘EFB.33’ and ‘Respect’, which were used as the resistant and susceptible control checks to root rot, respectively. The panel was grown in pots with naturally infested non-sterilized (NS) and sterilized soil (S) under controlled conditions in a growth chamber with four replicates. The fungal community of the infested soil was composed of several putative pea pathogens, including *Fusarium* spp., *Rhizoctonia solani*, and *Didymella* spp., as well as putative plant beneficial fungi such as *Clonostachys rosea*, *Coprinellus* sp. and several arbuscular mycorrhizal fungi [9, 26]. However, no method was applied for the identification of Oomycete species in the soil samples. Plant height and shoot dry weight (SDW) were measured to assess the overall performance of the plants in the trial. The traits plant emergence and root-rot index (RRI: 1 = no symptoms; 6 = complete disintegration of the root system) under NS conditions, as well as the shoot dry-weight ratio between NS and S conditions (SDW_NS/S_) were proposed to be used as surrogate measures to identify resistant genotypes. Plant emergence indicates resistance against damping-off, while RRI shows resistance of plants that managed to emerge, and SDW_NS/S_ indicates tolerance to disease damage [9]. In this study, we also included the ratio between root and shoot dry weight of the plants under NS conditions (RDW/SDW_NS_), which has been a useful indicator of root rot susceptibility in pea [27]. This trait is used in genetic and physiological studies as a measure of the partitioning of resources between roots for nutrient and water absorption, and shoots for light interception and photosynthesis. Broad sense heritabilities for each trait were calculated according to Wille et al. [9].

### Genotyping

Pea genotypes were grown from surface-sterilized seeds in sterile soil according to Wille et al. [9]. Approximately 0.25 cm^2^ frozen leaf tissue of two weeks old plants was milled with 2 mm ceramic beads for 60 s. Genomic DNA was isolated using the Mag-Bind® Plant DNA DS 96 Kit (Omega Bio-tek, Inc. Norcross, GA, USA) on a 96-well plate KingFisher Flex Purification System (Thermo Fischer Scientific, Waltham, MA, USA) following manufacturer recommendations. All samples were additionally cleaned using the DNA Clean & Concentrator® kit (Zymo Research, Irvine, CA, USA). DNA was visualized on a 1% agarose gel and quantified using a QIAxpert® spectrophotometer (QIAGEN Sciences, Germantown, MD, USA). The DNA was diluted to 20 ng/μl. Ten microliters of each sample were shipped in sealed 96-well plates on dry ice to Université Laval, Canada.

The population was genotyped-by-sequencing (GBS) following the protocol proposed by Poland et al. [28], using a combination of *Pst*I and *Msp*I as restriction enzymes. GBS libraries were prepared at the plateforme d’analyses génomiques of the Institut de Biologie Intégrative et des Systèmes (IBIS, Université Laval, Québec, Canada) with the following modifications: a BluePippin (Sage Scientific, Beverly, MA, USA) was used to size the libraries before PCR amplification (elution set between 50 and 65 min, on a 2% gel). Libraries were normalized, pooled, and then denatured in 0.02N NaOH and neutralized using HT1 buffer. Plate barcoding was used to enable sequencing on a shared Illumina NovaSeq S4 lane as described in Colston-Nepali et al. [29]. Sequencing was performed at the Centre d’expertise et de services Genome Québec in Canada. The pool was loaded at 225 pM on an Illumina NovaSeq S4 lane using the Xp protocol according to the manufacturer’s recommendations. The run was performed for 2 × 150 cycles (paired-end mode). A phiX library was used as a control and mixed with libraries at 1% level. Base calling was performed using RTA (RRID:SCR_014332; v3). The bcl2fastq2 software (RRID:SCR_015058; v2.20) was then used to demultiplex plates and generate FASTQ reads.

Sequence demultiplexing was performed with Stacks (RRID:SCR_003184; v2.60) [30], allowing up to one mismatch in the adapter sequence. Adapter tails were clipped with HTStream (RRID:SCR_018354; v1.3.3) (https://github.com/s4hts/HTStream). Using Bowtie (RRID:SCR_016368; v2.4.4) [31], the processed reads were mapped to the reference genomes of *P. sativum* cv. ‘Caméor’ (v1a, available at https://urgi.versailles.inra.fr/Species/Pisum as of November 2025) [19] and ‘Zhongwan 6’ (v1.0, available at https://www.peagdb.com/ as of November 2025) [20], hereafter referred to as Cam. and ZW6, respectively. The mapped reads were used for single nucleotide polymorphism (SNP) calling using NGSEP (RRID:SCR_012827; v4.1.0) [32]. The genotypic matrix was filtered for genotype calls with a quality score above 30, minor allele frequency (MAF) above 0.02 and a maximum observed heterozygosity rate of 0.05 per SNP marker. Finally, SNPs with less than 22% genotyped samples were removed to reduce the proportion of missing data in the genotypic matrix to approximately 30%. The predicted effect of these sequence variants on the gene models of the reference genomes was annotated with snpEff (RRID:SCR_005191; v5.0e) [33].

### Population structure and linkage disequilibrium

Population structure and linkage disequilibrium (LD) analyses were performed using the reference genome Cam. Pairwise measures of LD were calculated for each chromosome in sliding windows of 100 markers using the filtered genotypic matrix. The LD measures were corrected for kinship relationships in the population (${r}_{V}^{2}$) as implemented in the R package LDcorSV (v1.3.2) [34]. The LD decay was estimated regressing the pairwise ${r}_{V}^{2}$ values on the physical distance of their markers using the locally estimated scatterplot smoothing implemented in the R function ‘loess’ (v4.1.2), with a span value of 0.5. The population structure was assessed with a principal component analysis (PCA) using the genotypic matrix described above. Missing data in the matrix used for PCA was imputed using Beagle (RRID:SCR_001789; v5.2) [35], setting the effective population size to 1,000 and default parameters.

### Genome-wide association studies and genomic prediction

Association mapping was performed for each quantitative trait using the linear mixed model approach implemented in the R package GENESIS (v2.24.0) [36]. This model included three principal components and a kinship matrix to correct for population structure and relatedness. The association mapping on the binary trait leaf type was conducted using a generalized linear mixed model via the penalized quasi-likelihood approximation proposed by Chen et al. [37] and implemented in the same R package GENESIS. Significant associations were defined when the *p* value for each SNP marker was smaller than the Bonferroni-corrected threshold, which was calculated with a genome-wide type I error rate of 0.05.

A Bayesian ridge regression (BRR) model was fitted using the R package BGLR (v1.0.9) [38] with 10,000 iterations with a thin of 5, using 1,000 as burn-in. In this model, all imputed SNP markers were included as predictors of the phenotypic data, with a Gaussian prior assigned to marker effects. The genomic heritability was calculated using the remaining iterations thinned by a factor of 5. It used the sample variance of genomic values at each iteration of the sampler, as described by de los Campos et al. [39]. The estimates of marker effects were extracted from the fitted model. The prediction ability of the model was assessed by 50 times cross validation, randomly splitting the dataset into training (70%) and validation (30%) subsets. Pearson’s correlation coefficients between observed and predicted values of the validation subset were calculated to quantify the prediction ability for each trait. Marker densities and allele frequency filtering were tested to assess their impact on predictive abilities. Marker density was adjusted through LD pruning using the function ‘snpgdsLDpruning()’ of the SNPrelate R package (v1.42.0) [40], retaining markers with pairwise genotypic correlation (*r*^2^) below specified thresholds of 0.3, 0.5, 0.7, 0.9, 0.95 and 1 within sliding windows per chromosome. Allele frequencies were filtered to remove markers with MAF below 0.02, 0.05 and 0.1.

## Results

### Genotyping, linkage disequilibrium and population structure analysis

**Fig. 1:**
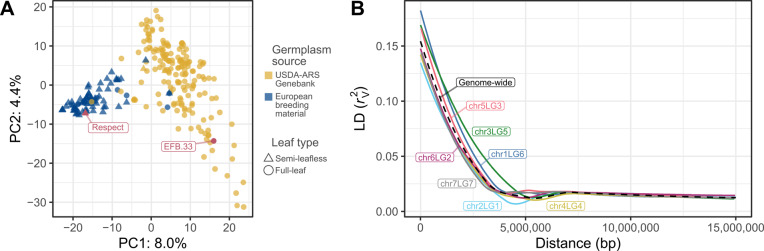
**A** Population structure assessment using principal component analysis of single nucleotide polymorphism (SNP) data on the reference genome of cv ‘Caméor’. The location of each genotype is represented by a point in the two-dimensional space defined by the first and second principal component (PC). The color of the points represents the germplasm source, while the shape represents their leaf type. The red tagged points show the location of the cv. ‘EFB.33’ and ‘Respect’, the resistant and susceptible control checks used in the growth chamber trial, respectively.** B** Patterns of linkage disequilibrium (LD) decay calculated genome-wide (black-dashed line) and for each chromosome separately (colored lines) from SNP data on the reference genome of cv. ‘Caméor’. Each line corresponds to a locally estimated scatterplot smoothing (LOESS) regression on the LD measures.

Genotyping-by-sequencing yielded a SNP matrix with 17,266 and 18,489 sequence variants using Cam. and ZW6 as reference genomes, respectively. The genotyping process failed for seven out of 261 accessions, which reduced the size of the population to 254 genotypes (Table S1). These variants were evenly distributed along the seven chromosomes of the two reference genomes (Figure S1). The population structure analysis of the SNP matrix revealed that the first and the second principal component accounted for 8.0% and 4.4% of the observed variance, respectively (Fig. 1A). The projection of genotypes in the bidimensional space defined by these principal components revealed two major groups that can mainly be distinguished by origin of the seed: the accessions sourced from the USDA-ARS gene bank and the European breeding material. These two groups were close to each other and contained a few accessions overlapping with the other group. Using pairwise measures of LD between the SNP markers, the genome-wide LD decay was 1.5 Mbp at 0.077 ${r}_{V}^{2}$, half of its maximum value (Fig. 1B). The LD decay rate was very similar among chromosomes, ranging between 1.2 Mbp at 0.091 ${r}_{V}^{2}$ (chr1LG6) to 1.53 Mbp at 0.067 ${r}_{V}^{2}$ (chr2LG1).

### Phenotypic correlations

High phenotypic correlations ranging between 0.64 and 0.92 were observed for the early vigor-related traits plant height and SDW, as published by Wille et al. [9] (Figure S2). These high correlations were consistent for trials managed under NS and S conditions. High heritabilities were observed for the early vigor-related traits, ranging between 0.92 to 0.98. On the other hand, the phenotypic correlations among root rot-related traits were inconsistent, ranging from |0.32| (emergence_NS_ vs. SDW_NS/S_) to |−0.60| (RRI_NS_ vs. SDW_NS/S_). The heritability for these traits was high only for plant emergence_NS_ (0.89), while the others showed lower values of 0.43 (RRI_NS_) and 0.51 (SDW_NS/S_). The correlations between early vigor and root rot-related traits did not show any clear pattern. They ranged between |0.46| (SDW_NS_ vs SDW_NS/S_) and |−0.54| (RRI_NS_ vs. SDW_NS_). The newly introduced variable RDW/SDW_NS_ did not show high correlations with any of the previously reported traits and had a heritability of 0.48.

### Association mapping

**Fig. 2:**
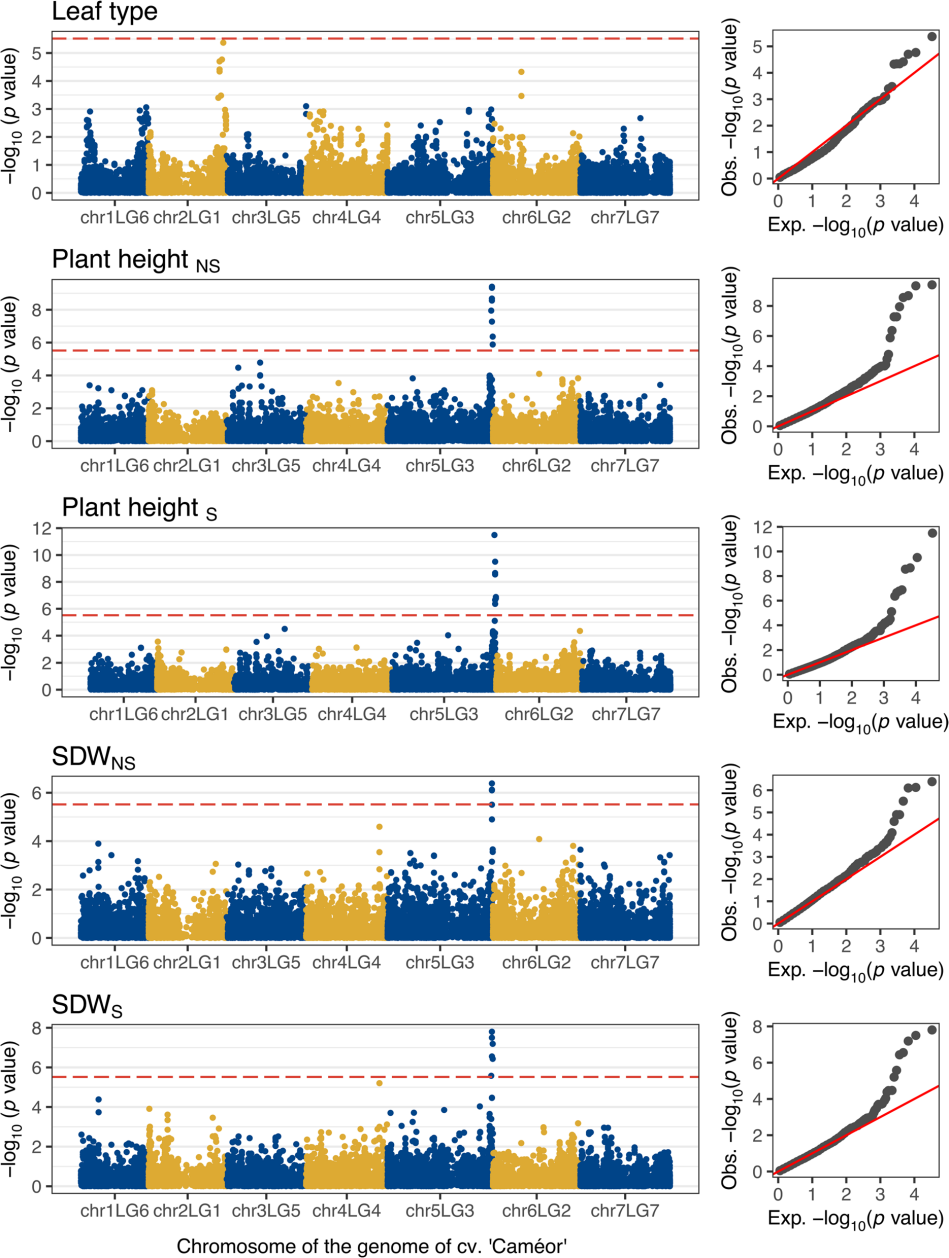
Results of the genome-wide association studies for the traits leaf type, plant height and shoot dry weight (SDW) under naturally infested (NS) or sterilized soil conditions (S). The horizontal, red-dashed line represents the Bonferroni-corrected threshold, which was calculated with a genome-wide type I error rate of α = 0.05 (*p* < 2.028 × 10^–6^). The results are presented as individual Manhattan plots showing the marker-trait association significance (*y* axis) of the single nucleotide polymorphisms (SNP) and their physical location on each of the seven chromosomes of the reference genome of cv. ‘Caméor’ (*x* axis). The corresponding quantile–quantile-plots to the right compare the deviation between the observed and the expected significance of the SNP from a theoretical *Χ*^2^ distribution.

The binary trait leaf type was associated with a single genomic region on chromosome chr2LG1, where the most significantly associated marker was located at 409,403,647 and 469,582,243 bp in the Cam. and ZW6 reference genomes, respectively (Fig. 2, Table 1, Figure S3). The *p* value of the markers PsCam_chr2LG1_409403647_G/A and PsZW6_chr2_469582243_G/A came close but did not reach the Bonferroni-corrected threshold of significance. A close examination of the distribution of this trait in the population revealed that the semi-leaf-less genotypes were almost exclusively present in the European breeding material (Fig. 1). This likely reduced the power to identify significant associations due to population structure correction.

**Table 1:** Genome-wide association results for the most significantly associated markers with leaf type, plant height, shoot and root dry weight (SDW and RDW, respectively), plant emergence and root rot index (RRI) evaluated under naturally infested or sterilized soil conditions (NS and S, respectively). The physical location of each marker is given by the chromosome (Chr.) and position (Pos.) in the reference genome of cv. ‘Caméor’ or ‘Zhongwan 6’. The level of significance (*p* value), the proportion of variance explained (PVE) and the estimated effect (Est. effect) of each marker were obtained from the corresponding mixed model used for association.

Trait	Ref. genome	SNP ID	Chr	Pos. (bp)	*p* value	PVE	Est. effect
Leaf type	Caméor	PsCam_chr2LG1_409403647_G/A	chr2LG1	409,403,647	4.26E-06	0.287	2.169
	Zhongwan 6	PsZW6_chr2_469582243_G/A	chr2	469,582,243	1.40E-05	0.269	2.092
Plant height_NS_	Caméor	PsCam_chr5LG3_573695584_C/A	chr5LG3	573,695,584	3.97E-10	0.157	4.641
	Zhongwan 6	PsZW6_chr5_646378981_T/C	chr5	646,378,981	9.39E-10	0.150	5.325
Plant height_S_	Caméor	PsCam_chr5LG3_569788697_G/T	chr5LG3	569,788,697	3.25E-12	0.194	6.393
	Zhongwan 6	PsZW6_chr5_642030534_C/A	chr5	642,030,534	2.52E-13	0.214	6.867
SDW_NS_	Caméor	PsCam_chr5LG3_573520009_A/C	chr5LG3	573,520,009	4.13E-07	0.103	0.021
	Zhongwan 6	PsZW6_chr5_646205699_A/C	chr5	646,205,699	1.10E-06	0.095	0.021
SDW_S_	Caméor	PsCam_chr5LG3_573520009_A/C	chr5LG3	573,520,009	1.58E-08	0.128	0.024
	Zhongwan 6	PsZW6_chr5_646205699_A/C	chr5	646,205,699	1.19E-09	0.148	0.027
RRI_NS_	Caméor	PsCam_chr6LG2_68264764_T/C	chr6LG2	68,264,764	2.73E-06	0.088	−0.210
	Zhongwan 6	PsZW6_chr6_85080347_T/C	chr6	85,080,347	4.32E-06	0.084	−0.213
SDW_NS/S_	Caméor	PsCam_chr6LG2_68264779_G/T	chr6LG2	68,264,779	5.11E-07	0.101	0.092
	Zhongwan 6	PsZW6_chr6_85080362_G/T	chr6	85,080,362	1.07E-07	0.113	0.100
Plant emergence_NS_	Caméor	PsCam_chr6LG2_68269898_A/T	chr6LG2	68,269,898	2.07E-09	0.157	0.138
	Zhongwan 6	PsZW6_chr6_85085481_A/T	chr6	85,085,481	6.80E-09	0.147	0.133

**Fig. 3:**
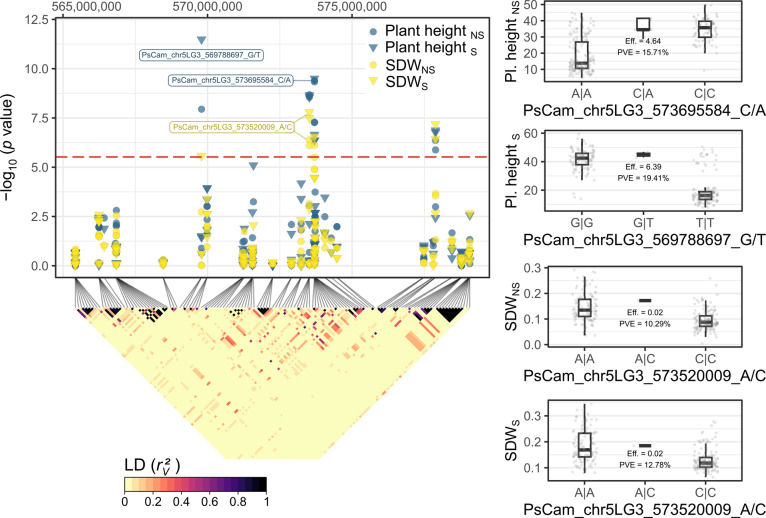
Genetic dissection of the region of association on chromosome chr5LG3 for the traits plant height and shoot dry weight (SDW) under naturally infested (NS) and sterilized (S) soil conditions. The scatterplot shows combined results of individual genome-wide association studies (GWAS). Each point shows the marker-trait association significance (*y* axis) of each single nucleotide polymorphism (SNP) and their physical location on chr5LG3 in the genome of cv. ‘Caméor’ (*x* axis). The horizontal, red-dashed line represents the Bonferroni-corrected threshold, which was calculated with a genome-wide type I error rate α = 0.05 (*p* < 2.028 × 10^–6^). The tagged points indicate the most significantly associated SNPs for each trait. The colored square matrix below represents the pairwise linkage disequilibrium (LD) measurements (*r*_*v*_^2^) between each pair of SNPs in the region of association. The boxplots show the distribution of phenotypic values between the genotypes of the most significantly associated SNPs on chr5LG3. Each plot includes the marker effect (Eff.) and the proportion of variance explained (PVE) derived from the GWAS model.

The early vigor traits plant height and SDW under NS and S conditions were associated with a single region in the distant arm of chromosome chr5LG3 between 565–580 Mbp and 637–660 Mbp in the Cam. and ZW6 reference genomes, respectively (Fig. 2, Table 1 and Figure S3). Closer examination of this region revealed at least three peaks in the associated genome region (Fig. 3). The most significantly associated markers for plant height defined the first two subregions of association at 569,788,697 bp and 573,695,584 bp in the Cam., and 642,030,534 bp and 646,378,981 bp in the ZW6 reference genome. The estimated marker effects ranged from 4.64 to 6.87, and the proportion of variance explained (PVE) from 0.150 to 0.214 (Fig. 3, Table 1, Figure S4; table with full association results is provided in the Data availability section). The SNPs PsCam_chr5LG3_569788697_G/T and PsZW6_chr5_642030534_C/A were located at the intron region of the gene models Psat5g301400_Cam._ | Psat05G0828800_ZW6_, which encode a member of the Nucleoporin interacting component (Nup93/Nic96-like) family (74% sequence identity to AT2G41620.1 in *Arabidopsis thaliana*). The SNPs PsCam_chr5LG3_573695584_C/A and PsZW6_chr5_646378981_T/C were located at the intron region of the gene models Psat5g304720_Cam._ | Psat05G0837000_ZW6_, which encode a homologue of the Golgi SNARE 11 protein (GOS11; 80% sequence identity to AT1G15880.1 in *A. thaliana*). The most significantly associated markers for SDW_S_ and SDW_NS_ PsCam_chr5LG3_573520009_A/C and PsZW6_chr5_646205699_A/C were located within the second subregion of association, with an estimated effect between 0.02–0.03 g and PVE between 0.095–0.148 (Fig. 3, Table 1 and Figure S4). The third subregion was shared by all four traits and included the significantly associated markers PsCam_chr5LG3_577882635_T/C | PsZW6_chr5_652345014_T/C and PsCam_chr5LG3_577882853_A/G | PsZW6_chr5_652345232_A/G, with estimated effects between 3.14–4.44 cm and 0.012–0.022 g, and a PVE between 0.068–0.111 and 0.034–0.117, respectively (table with full association results is provided in the Data availability section).

**Fig. 4:**
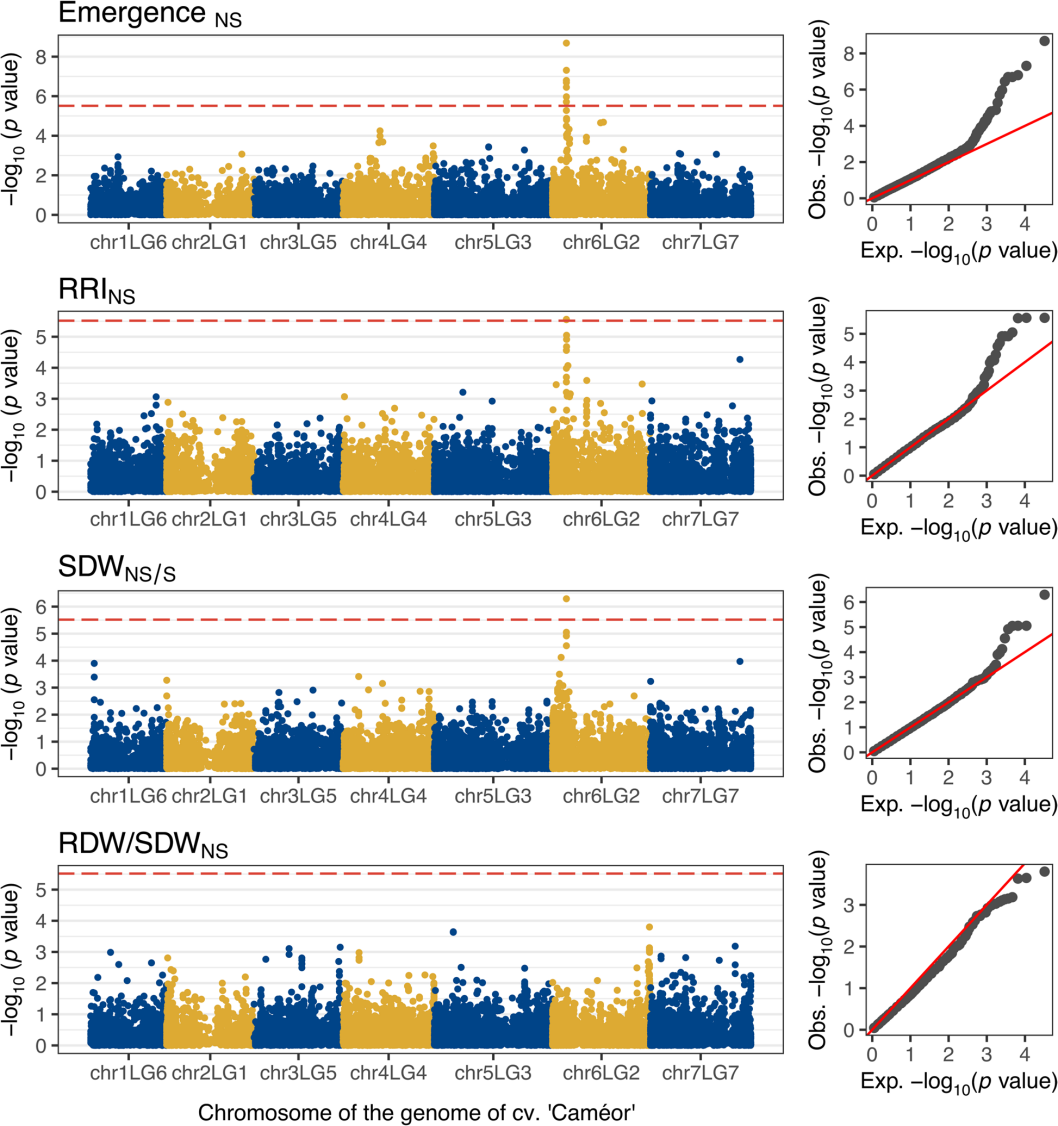
Results of the genome-wide association studies for the root rot-related traits plant emergence and root rot index (RRI) under naturally infested soil conditions (NS), shoot dry weight (SDW) ratio between naturally infested and sterilized soil conditions (NS/S), and the ratio between root and shoot dry weight (RDW/SDW) under NS conditions. The horizontal, red-dashed line represents the Bonferroni-corrected threshold, which was calculated with a genome-wide type I error rate α = 0.05 (*p* < 2.028 × 10^–6^). The results are presented as individual Manhattan plots showing the marker-trait association significance (*y* axis) of the single nucleotide polymorphisms (SNP) and their physical location on each of the seven chromosomes of the reference genome of cv. ‘Caméor’ (*x* axis). The corresponding quantile–quantile-plots to the right compare the deviation between the observed and the expected significance of the SNP from a theoretical *Χ*^2^ distribution.

**Fig. 5:**
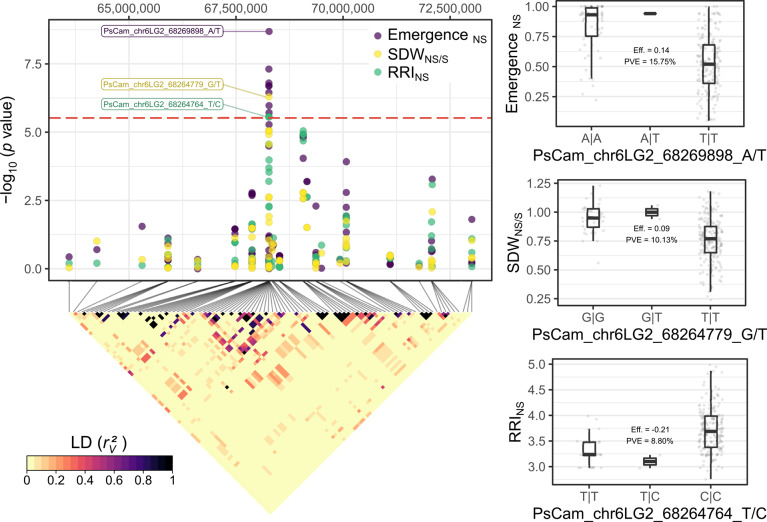
Genetic dissection of the region of association on chromosome chr6LG2 for the root rot-related traits plant emergence, root rot index (RRI) and shoot dry weight (SDW) under naturally infested (NS) and sterilized (S) soil conditions. The scatterplot shows combined results of individual genome-wide association studies (GWAS). Each point shows the marker-trait association significance (*y* axis) of each single nucleotide polymorphism (SNP) and their physical location on chr6LG2 in the genome of cv. ‘Caméor’ (*x* axis). The horizontal, red-dashed line represents the Bonferroni-corrected threshold, which was calculated with a genome-wide type I error rate α = 0.05 (*p* < 2.028 × 10^–6^). The tagged points indicate the most significantly associated SNPs for each trait. The colored square matrix below represents the pairwise linkage disequilibrium (LD) measurements (*r*_*v*_^2^) between each pair of SNPs in the region of association. The boxplots show the distribution of phenotypic values between the genotypes of the most significantly associated SNPs on chr6LG2. Each plot includes the marker effect (Eff.) and the proportion of variance explained (PVE) derived from the GWAS model.

The root rot-related traits plant emergence_NS_, RRI_NS_ and SDW_NS/S_ were concordantly associated with a single region in the proximal arm of chromosome chr6LG2. However, the newly introduced trait RDW/SDW_NS_ was not associated with any region of the genome (Fig. 4, Table 1 and Figure S5). All significantly associated markers for these traits were in strong LD within a region spanning approximately 10 kbp around 68.265 Mbp and 85.085 Mbp in the Cam. and ZW6 reference genomes, respectively (Fig. 5). The estimated effects for the most significantly associated markers for SDW_NS/S_, RRI_NS_ and emergence_NS_ were 0.100, −0.210 and 0.135 and their PVE was 0.10, 0.08 and 0.15, respectively (Fig. 5, Table 1, and Figure S6). This region co-locates with the gene models Psat6g060320_Cam._ | Psat06G0169300_ZW6_, which encode a *mitochondrial Rho* (*MIRO*)-related GTPase. The markers PsCam_chr6LG2_68264764_T/C | PsZW6_chr6_85080362_G/T and PsCam_chr6LG2_68264779_G/T | PsZW6_chr6_85080347_T/C fall within the intron region of Psat6g060320_Cam._ | Psat06G0169300_ZW6_, whereas the annotation of PsCam_chr6LG2_68269898_A/T | PsZW6_chr6_85085481_A/T predicts a synonymous mutation in the exon region of the same gene models (table with full association results is provided in the Data availability section).

### Genomic prediction

**Fig. 6:**
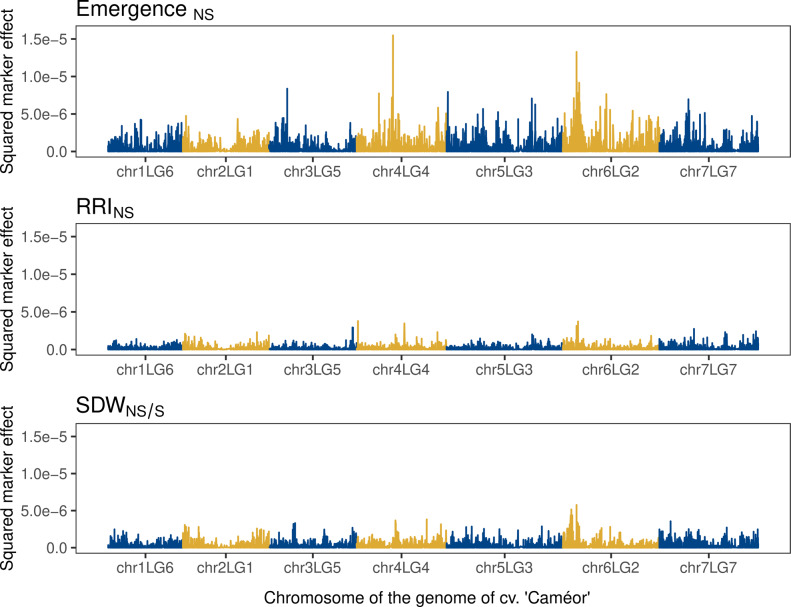
Single nucleotide polymorphism (SNP) effects derived from a Bayes ridge regression model that used all SNP markers as predictors of root rot-related traits: plant emergence and root rot index (RRI) under naturally infested soil conditions (NS), and shoot dry weight (SDW) ratio between naturally infested and sterilized soil conditions (NS/S). The squared marker effects (*y* axis) are plotted along their physical position on each one of the seven chromosomes of the reference genome of cv. ‘Caméor’ (*x* axis)

The whole-genome regression model yielded variable results consistent with the broad sense heritability of each trait, but unaffected by the reference genome used (Table 2). The genomic heritabilities ranged between 0.25 (RRI_NS_) and 0.84 (plant height_S_). Similarly, the mean prediction ability of the BRR model ranged between 0.13 (RRI_NS_) and 0.81 (plant height_S_). The missing heritability, defined as the difference between broad sense and genomic heritability, showed less variation with values ranging between 0.14 (plant height_S_) and 0.36 (emergence_NS_). The estimated marker effects for root rot-related traits were larger for emergence_NS_ (Fig. 6 and Figure S7). In line with the results of the genome-wide association studies (GWAS), the BRR model showed a SNP marker with a large effect on chr6LG2 at 68.265 Mbp and 85.085 Mbp in the Cam. and ZW6 reference genomes, respectively. However, it also showed a SNP on chr4LG4 with a comparable marker effect at 180.86 Mbp and 216.78 Mbp in the Cam. and ZW6 reference genomes, respectively. This region coincides with a non-significant peak in chr4LG4 observed for emergence_NS_ (Fig. 4 and Figure S5). Other markers on chr3LG5, chr6LG2 and chr7LG7 showed minor effects for emergence_NS_ (Fig. 6 and Figure S7). Predictive abilities were unaffected by marker density and allele frequency filtering (Figure S8). Taken together, all 17,266 and 18,489 SNP markers explained up to 51% and 53% of the observed phenotypic variance for emergence_NS_ in the Cam. and ZW6 reference genomes, respectively (Table 2).

**Table 2:** Summary of the broad sense, genomic and missing heritabilities (H^2^), prediction abilities from the whole-genome regression model and proportion of variance explained (PVE) by the most significantly associated marker from the genome-wide association model (GWAS) for plant height, shoot and root dry weight (SDW and RDW), plant emergence and root rot index (RRI) under naturally infested (NS), sterilized soil conditions (S), or the ratio between NS/S conditions. These results were obtained using the reference genome of cv. ‘Caméor’ and ‘Zhongwan 6’. The mean and standard deviation (SD) of prediction abilities were calculated from Pearson’s correlation coefficients between observed and predicted values of the validation subset (30%) in a cross-validation process repeated 50 times.

Trait	Broad sense heritability*	Ref. genome	Genomic heritability	Missing heritability	Prediction ability (mean ± SD)	PVE GWAS
Plant height_NS_	0.96	Caméor	0.80	0.16	0.77 ± 0.04	0.16
		Zhongwan 6	0.81	0.15	0.77 ± 0.04	0.15
Plant height_S_	0.98	Caméor	0.84	0.14	0.79 ± 0.04	0.19
		Zhongwan 6	0.83	0.15	0.81 ± 0.03	0.21
SDW_NS_	0.92	Caméor	0.60	0.32	0.54 ± 0.07	0.10
		Zhongwan 6	0.63	0.29	0.52 ± 0.06	0.10
SDW_S_	0.92	Caméor	0.72	0.20	0.67 ± 0.05	0.13
		Zhongwan 6	0.72	0.20	0.70 ± 0.05	0.15
SDW_NS/S_	0.51	Caméor	0.30	0.21	0.25 ± 0.11	0.10
		Zhongwan 6	0.34	0.17	0.27 ± 0.10	0.11
RRI_NS_	0.43	Caméor	0.25	0.18	0.13 ± 0.09	0.09
		Zhongwan 6	0.26	0.17	0.13 ± 0.09	0.08
Plant emergence_NS_	0.89	Caméor	0.51	0.35	0.49 ± 0.07	0.16
		Zhongwan 6	0.53	0.36	0.51 ± 0.08	0.15
RDW/SDW_NS_	0.48	Caméor	0.33	0.15	0.27 ± 0.10	•
		Zhongwan 6	0.34	0.14	0.33 ± 0.09	•

## Discussion

### A novel QTL and candidate genes for resistance to root rot in pea

Resistance to root rot in pea is of quantitative nature and has been studied for decades, resulting in the identification of multiple QTL across the genome [3]. In this study, a single genomic region was consistently associated with the root rot-related traits on chromosome chr6LG2 at 68.265 Mbp and 85.085 Mbp in the Cam. and ZW6 reference genomes, respectively. Other QTL for root rot resistance in pea have been reported on this chromosome: First, the moderate effect QTL *Ae*_*MRCD1*_*Ps-2.1* and *Ae*_*MRCD1*_*Ps-2.2* against *A. euteiches* were identified by Wu et al. [23] between 404.587 and 465.936 Mbp (Cam.), with R^2^ values of 14.3% and 13.8%, respectively. Second, the QTL *Ae-Ps2.1* and *Ae-Ps2.2* on chr6LG2 showed R^2^ values of 15.4% and 26.9%, respectively [41, 42]. However, the exact physical location of these QTL was not available in any of Cam. or ZW6 reference genomes (Pulse Crop Database,www.pulsedb.org; as of November 2025). Third, the QTL *Ae-Ps2.4* with R^2^ of 5.8% was recently mapped to a region around 103,045 Mbp (Cam.) [43]. Finally, the resistance QTL *Fsp-Ps2.1* against *F. solani* was consistently identified on this chromosome by Coyne et al. [44, 45] with R^2^ values between 57.1% and 72.2%. The neighboring markers to *Fsp-Ps2.1* are Ps900203 and Ps000075 [45–47], which map to a region around 235,636 Mbp (Cam.; Pulse Crop Database,www.pulsedb.org; as of November 2025). The different physical locations of these markers suggest that these QTL are independent of each other. Taken together, our results indicate a narrow region on chr6LG2 that is not linked to any of these previously reported QTL, which is consistent with the quantitative nature of resistance.

A few candidate genes have been proposed from the identification of multiple QTL for resistance to root rot in legumes. For instance, three defensin genes were mapped together near the QTL *Fsp-Ps3.1* identified on chr5LG3 [44]. These genes were upregulated upon *F. solani* infection in pea [48]. Similarly, an *F-box* encoding gene (Medtr3g011020) was identified in the resistance of *Medicago truncatula* to *A. euteiches* [49]. More recently, a list of 39 candidate genes from transcriptomic analysis for *Aphanomyces* resistance was reported. These genes are distributed over all chromosomes, but the most promising were located on chr7LG7 of the Cam. reference genome [25]. Despite these examples, the molecular mechanisms underlying resistance to root rot in legumes are poorly understood to date [16, 18]. In this study, the region of association on chromosome chr6LG2 coincides with the gene models Psat6g060320_Cam._ | Psat06G0169300_ZW6_ that encode a *MIRO*-related GTPase. Its peptide sequence is homologous to the gene models Lcu.2RBY.1g063750.1 (*Lens culinaris*), Medtr1g072280.1 (*M. truncatula*), and *MIRO1* (AT5G27540.1; *Arabidopsis thaliana*), with 95%, 83% and 54% sequence identity, respectively [50–52].

Research on plant *Rho* GTPases is still in its infancy. Yet, increasing evidence indicates that they play a critical role in plant immunity [53–55]. For example, silencing of the *M. truncatula Rho of plant 9* (*MtROP9*) gene favored early root colonization by mycorrhizal fungi and *A. euteiches* [56]. Consistent with these findings, the *MIRO-*related *GTPase* Medtr1g072280 was proposed as a candidate tolerance gene against the root lesion nematode *Pratylenchus neglectus* in *Medicago littoralis* [57]. Despite these examples, some studies have shown that the homologue genes *MIRO1* (AT5G27540) and *MIRO2* (AT3G63150) in *Arabidopsis* are involved in salt stress response, embryogenesis, pollen tube growth and mitochondrial development [58–61]. In this sense, the mechanism by which a mitochondrial GTPase might be involved in root rot resistance is undetermined. Although the *MIRO-*related *GTPases* Psat6g060320_Cam._ | Psat06G0169300_ZW6_ have been consistently identified for three root rot-related traits in this study, it is still possible that the causal gene(s) or mutation(s) lie(s) in the surrounding region. Indeed, the gene models Psat6g063320_Cam._ | Psat06G0173100_ZW6_ are the pea homologues of the *F-box* gene (Medtr3g011020) identified in *M. truncatula* resistance to *A. euteiches* [49]. This gene is located 2.22 Mbp downstream of the association region on chr6LG2 and is also a plausible candidate. Similarly, the defense response candidate genes Psat6g042720_Cam._, Psat6g042840_Cam._ and Psat6g043800_Cam._ identified by Kälin et al. [25] are located approximately 30 Mbp upstream of the association region containing the *MIRO*-related *GTPases*.

### The quantitative nature of resistance to root rot is supported by multiple QTL

Whole-genome regression and GWAS models, despite their similarities, serve different purposes in breeding [62, 63]. Our GWAS results point to a single major QTL in chr6LG2, which accounts for a moderate proportion of the phenotypic variability for root rot-related traits. Given the genotypic variation and inconsistent phenotypic correlations between these traits, suggesting different genetic control mechanisms for resistance [9], we used a whole-genome regression model to supplement GWAS results. This model showed subtle marker effects in the region of association in chr6LG2 for RRI_NS_ and SDW_NS/S_ which limits conclusions about their genetic control. However, markers with the highest estimated effects for plant emergence_NS_, the disease resistance trait with the highest heritability, coincide with the region of association on chr6LG2. Comparable marker effects were also found on chr4LG4 at 180.86 Mpb (Cam.) and 216.78 Mbp (ZW6), with additional minor effects markers observed on chr6LG2, chr3LG5 and chr7LG7. The variance explained by all 18k markers (0.51_Cam._ – 0.53_ZW6_) exceeds that of the most significantly associated markers identified by GWAS (0.16_Cam._ – 0.15_ZW6_), highlighting the contribution of multiple QTL with moderate to small effects. Notably, the region on chr4LG4 is near the moderate-effect QTL *Fg-Ps4.1* (202,652,401–234,329,355 bp in Cam.) and *Fg-Ps4.2* (184,181,231–194,948,669 bp in Cam.) reported against *F. graminearum* [24]. A major-effect QTL, *Ae-Ps4.5,* was recently fine-mapped to a 3.06 Mbp interval in chr4LG4 (296,700,000–299,750,000 bp in Cam.) [64]. Additional minor resistance QTL have also been reported on chr3LG5, chr4LG4, chr6LG2 and chr7LG7 [3, 23, 45, 65]. Furthermore, the whole-genome regression model achieved a predictive ability of up to 0.51 for plant emergence_NS_. While this could be useful for designing genomic selection strategies, further validation using cross-environment schemes or independent validation sets is needed to fully realize its application to improve resistance to root rot in a pea breeding program [4]. Similar approaches have already shown promise for root rot resistance in common bean [66].

### Early vigor QTL were related to the *Le* locus, not root rot resistance

Plant height is a morphological trait for which high heritabilities and predictive abilities have been reported in pea [9, 23, 67]. We identified a genomic region between 565–580 Mbp and 637–660 Mbp on chr5LG3 in the Cam. and ZW6 reference genomes, respectively, associated with plant height and SDW under NS and S conditions. Several studies reported the identification of QTL associated with plant height on chr5LG3. These include the QTL *HT-Ps3.1*, located at 493.071 Mbp in the Cam. reference genome [42]; the QTL *WB.FspPs5.2* and *WB.FspPs5.1*, located between 516–569 Mbp and 236–296 Mbp of the Cam. reference genome, respectively [22]; and a QTL from a GWAS located between 566–573 Mbp in the same genome [68], which is consistent with the results presented in this study. In line with our results for SDW, Burstin et al. [69] and Klein et al. [70] reported QTL in chr5LG3 that are associated both with reduced plant height and vegetative biomass, whereas Wu et al. [23] found no association on chr5LG3 for dry foliage weight. Some of these studies link the identified QTL to the well-known *Le* locus that controls internode length in pea [71–73]. This locus encodes a gibberellin 3β-hydroxylase that controls the 3β-hydroxylation of gibberellin A_20_ to gibberellin A_1_ [74]. A BLAST search on the peptide sequence of this protein reveals that it corresponds to the gene models Psat5g299720_Cam._ | Psat05G0825300_ZW6_. These models are located approx. 2.42 Mbp upstream of the most significantly associated markers for plant height PsCam_chr5LG3_569788697_G/T | PsZW6_chr5_642030534_C/A identified in this study. Various surrogate traits have been used to evaluate root rot in pea plants under controlled conditions, such as disease severity-related indices, biomass of shoot and root sections of the plant, or plant height and vigor [27, 65, 75]. The results presented in this study show that early vigor and root rot-related traits have independent associations on chromosomes chr5LG3 and chr6LG2, respectively. These observations match previous findings, showing that plant height is a poor measure of root rot severity in pea [23, 24], while they also challenge other reports where plant height was more closely related to disease severity [22, 75]. In parallel, our results highlight the caution in using SDW (i.e. SDW_NS_ or SDW_S_) as a root rot-related trait, as it showed stronger relationships with plant height but not disease resistance. Instead, RRI_NS_, SDW_NS/S_ and plant emergence_NS_ provided a predictable measurement of the plant health [9], yielding consistent associations in the genome. Finally, the newly introduced root-shoot ratio trait RDW/SDW_NS_ was reported to be a useful indicator of plant susceptibility to root rot [27]. In this study, it presented moderate broad sense heritability and low phenotypic correlation with root rot-related traits. These characteristics made it a suitable prospect to further elucidate the genetic basis controlling the resistance against root rot. However, no significant association was observed for this trait which undermined its potential as a surrogate of plant health.

### The *afila* locus is not related to root rot resistance in pea

In pea, the semi-dwarf, semi-leafless type is characterized by superior standing ability, creates less favorable conditions for pests and foliar diseases and maintains yield performances comparable to those of full-leaf types [76, 77]. These features make it an important achievement of pea breeding [78]. The semi-leafless type was obtained by the introduction of the *afila* (*af*) mutation with retained full-type stipules. *af* has been studied for several decades and is reported on multiple genetic linkage maps [79]. In this study, the presence-absence of semi-leafless types in the population was associated to a region in the distant arm of chr2LG1, at 409 Mbp and 469 Mpb in the Cam. and ZW6 reference genomes, respectively, confirming recent reports [72, 73]. This region is 3 Mbp downstream of a region containing two PALMATE-LIKE PENTAFOLIATA 1 s (*PsPALM1a* and *PsPALM1b*), whose deletion is reported to be associated to the *af* mutation [80, 81]. Previous reports have shown that a DNA marker for *af* segregated with the resistance QTL *Ae-Ps1.2*, where the resistance allele is in coupling phase with the full-leaf type allele [41]. In fact, this allele was later proposed as a pleiotropy marker affecting leaf morphology and *Aphanomyces* resistance [42]. However, we did not find any association on chr2LG1 for root rot-related traits.

## Conclusions

In this work, we identified a novel QTL for resistance to root rot caused by a pathogenic complex present in infested soil. We used data from a diverse collection of pea genotypes grown in naturally infested soil. This approach brings experimental and on-farm conditions in the soil closer together, facilitating their application in real breeding contexts. Our results provide a promising opportunity for implementing marker-assisted and genomic selection strategies to improve root rot resistance in pea breeding schemes. While this study considered the effect of the entire microbiome and pathobiome, the analyses to identify genomic regions associated with resistance to root rot focused only on plant traits. However, the microbiome composition may substantially contribute to the ability to predict resistance. Further research should address the effect of the rhizosphere microbial community on root rot. This could provide additional tools that can be incorporated into the selection process in a breeding program.

## Supplementary Information

Supplementary Material 1: Figure S1. Heatmap showing SNP density across the seven chromosomes of two pea reference genomes, highlighting centromeric regions. Figure S2. Correlation matrix of plant vigor and root rot-related traits and their heritabilities. Figure S3. Manhattan and Q-Q plots of GWAS results for leaf type, plant height, and shoot dry weight using the reference genome of cv. 'Zhongwan 6'. Figure S4. Detailed GWAS analysis of chromosome chr5 for early vigor traits using the reference genome of cv. 'Zhongwan 6'. Figure S5. Manhattan and Q-Q plots of GWAS results for root rot-related traits using the reference genome of cv. 'Zhongwan 6'. Figure S6. Detailed GWAS analysis of chromosome chr6 for root rot resistance traits using the reference genome of cv. 'Zhongwan 6'. Figure S7. Distribution of squared SNP effects from Bayesian ridge regression across the seven chromosomes of the reference genome of cv. 'Zhongwan 6' for root rot-related traits. Figure S8. Prediction ability under varying marker densities and MAF thresholds for root rot-related traits using the reference genomes of cv. ‘Caméor’ and ‘Zhongwan 6’.

Supplementary Material 2: Table S1. List of plant material and phenotypic data used in this study.

## Data Availability

Raw sequencing data for the 254 pea genotypes are deposited in the NCBI Sequence Read Archive (SRA) under BioProject number PRJNA1127519. The genotypic matrices generated in this study using the Cam. and ZW6 reference genomes are deposited at the ETH Research Collection under 10.3929/ethz-b-000681298. These matrices are provided in compressed Variant Call Format (VCF) and Genomic Data Structure (GDS) formats. The phenotypic data used in this study were originally reported by Wille et al. [9] and is reproduced here in Table S1. Detailed association results for leaf type, plant vigor and root rot-related traits, together with the phenotypic data, were deposited in the same entry of the ETH Research Collection for reference.

## Abbreviations

BRR: Bayesian Ridge Regression
Cam.: Reference genome of cv. ‘Caméor’
GBS: Genotyping-by-Sequencing
GWAS: Genome-Wide Association Studies
LD: Linkage Disequilibrium
NS/S: Ratio of trait values under NS and S conditions
NS: Naturally Infested Soil
PCA: Principal Component Analysis
PVE: Proportion of Variance Explained
QTL: Quantitative Trait Locus / Quantitative Trait Loci
RDW/SDW_NS_: Ratio of RDW to SDW under NS
RDW: Root Dry Weight
RRI: Root Rot Index
S: Sterilized Soil
SDW: Shoot Dry Weight
SDW_NS/S_: Ratio of SDW under NS to S
SDW_NS_: Shoot Dry Weight under NS
SDW_S_: Shoot Dry Weight under S
SNP: Single Nucleotide Polymorphism
USDA-ARS: United States Department of Agriculture - Agricultural Research Station
ZW6: Reference genome of cv. ‘Zhongwan 6
