# Supplementary material for: Association genetics and genomic prediction for resistance to root rot in a diverse collection of Pisum sativum L
Source: BMC Plant Biol. 2025 Dec 19;26:131. doi: 10.1186/s12870-025-07803-0 (PMC12831431; doi:10.1186/s12870-025-07803-0)
Supplement: Supplementary file 1 — Supplementary Material 1: Figure S1. Heatmap showing SNP density across the seven chromosomes of two pea reference genomes, highlighting centromeric regions. Figure S2. Correlation matrix of plant vigor and root rot-related traits and their heritabilities. Figure S3. Manhattan and Q-Q plots of GWAS results for leaf type, plant height, and shoot dry weight using the reference genome of cv. 'Zhongwan 6'. Figure S4. Detailed GWAS analysis of chromosome chr5 for early vigor traits using the reference genome of cv. 'Zhongwan 6'. Figure S5. Manhattan and Q-Q plots of GWAS results for root rot-related traits using the reference genome of cv. 'Zhongwan 6'. Figure S6. Detailed GWAS analysis of chromosome chr6 for root rot resistance traits using the reference genome of cv. 'Zhongwan 6'. Figure S7. Distribution of squared SNP effects from Bayesian ridge regression across the seven chromosomes of the reference genome of cv. 'Zhongwan 6' for root rot-related traits. Figure S8. Prediction ability under varying marker densities and MAF thresholds for root rot-related traits using the reference genomes of cv. ‘Caméor’ and ‘Zhongwan 6’. [file 12870_2025_7803_MOESM1_ESM.docx]

Association genetics and genomic prediction for resistance to root rot in a diverse collection of *Pisum sativum* L.

Daniel Ariza-Suarez^1^, Lukas Wille^1,2^, Pierre Hohmann^2,3^, Valentin Gfeller^2^, Michael Schneider^2^, Matthew W. Horton^2^, Monika M. Messmer^2*^, Bruno Studer^1*^

^1^Molecular Plant Breeding, Institute of Agricultural Sciences, ETH Zurich, Zurich, Switzerland.

^2^Department of Crop Sciences, Research Institute of Organic Agriculture (FiBL), Frick, Switzerland.

^3^Department of Biology, Healthcare and the Environment, Faculty of Pharmacy and Food Sciences, Universitat de Barcelona, 08028 Barcelona, Spain.

**^*^Correspondence:** [bruno.studer@usys.ethz.ch](mailto:bruno.studer@usys.ethz.ch) - [monika.messmer@fibl.org](mailto:monika.messmer@fibl.org)

Author ORCIDs

| Daniel Ariza-Suarez | 0000-0002-1871-3514 |
| --- | --- |
| Lukas Wille | 0009-0004-4090-3826 |
| Pierre Hohmann | 0000-0001-7029-0566 |
| Valentin Gfeller | 0000-0001-8896-7280 |
| Michael Schneider | 0000-0002-6491-8852 |
| Matthew W. Horton | 0000-0002-7537-0730 |
| Monika M. Messmer | 0000-0002-6120-0079 |
| Bruno Studer | 0000-0001-8795-0719 |

# Additional Files


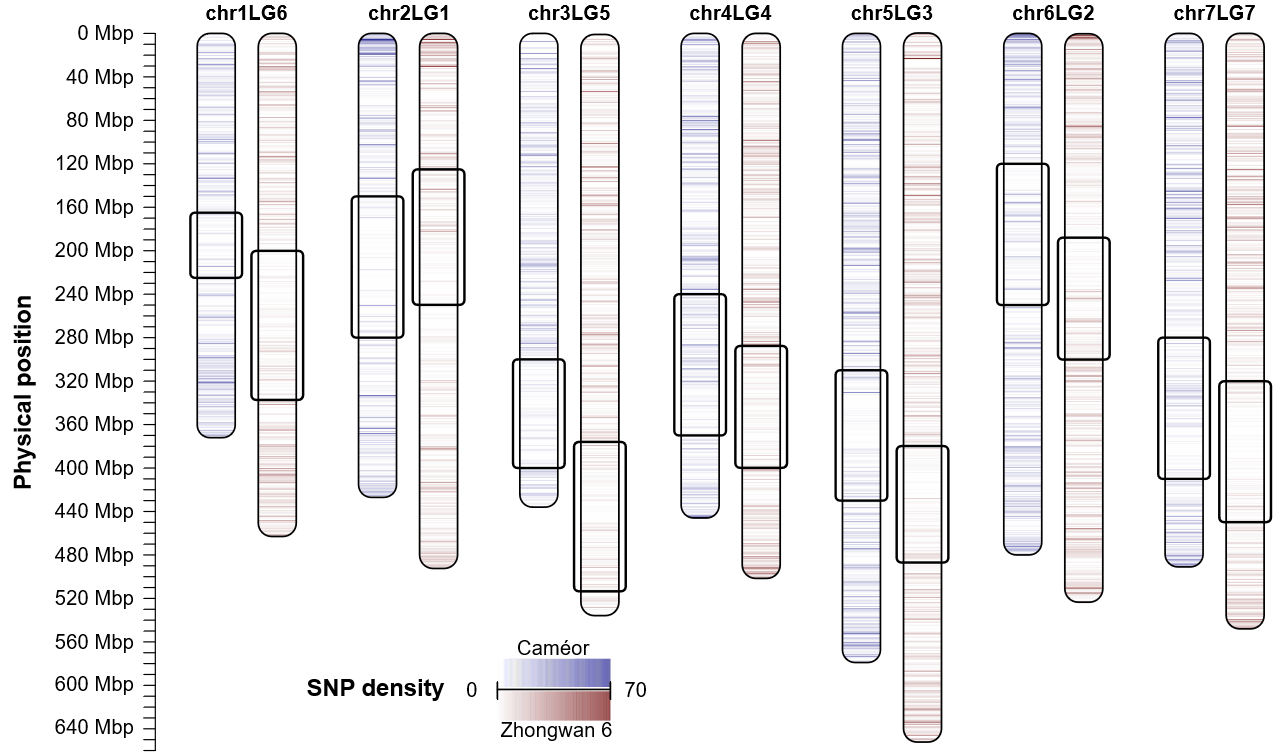


Figure S1. Heatmap for the density of single nucleotide polymorphisms (SNPs) along the seven chromosomes of the reference genomes of *Pisum sativum* L. cv. ‘Caméor’ (blue) and ‘Zhongwan’ 6 (red), identified by genotyping-by-sequencing. Each color band represents a region of 1 Mbp and its color intensity represents the SNP density. The inner black rectangles on each chromosome represent the boundaries of the centromeric regions as defined by Kreplak et al. (2019) and Yang et al. (2022).


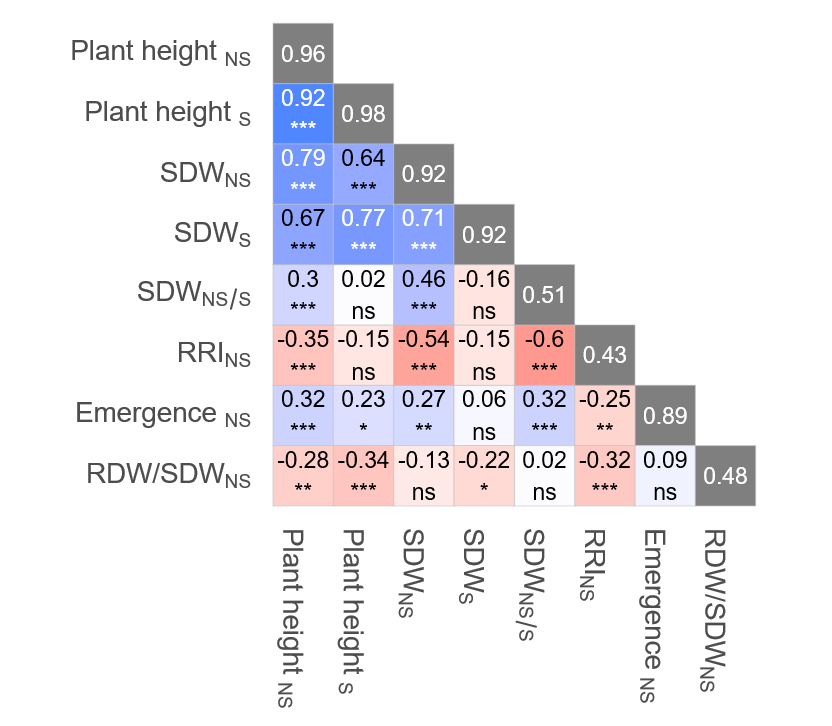


Figure S2. Phenotypic correlations between adjusted means of the traits plant height, shoot and root dry weight (SDW and RDW), plant emergence and root rot index (RRI) under naturally infested (NS), sterilized soil conditions (S), the ratio between NS/S conditions, and the ratio between root and shoot dry weight (RDW/SDW) under NS conditions. The broad sense heritabilities are indicated within the main diagonal with gray background for each trait. Significance of correlations indicated as ***: *p* < .0001; **: *p* < .001; *: *p* < .01; ns: not significant.


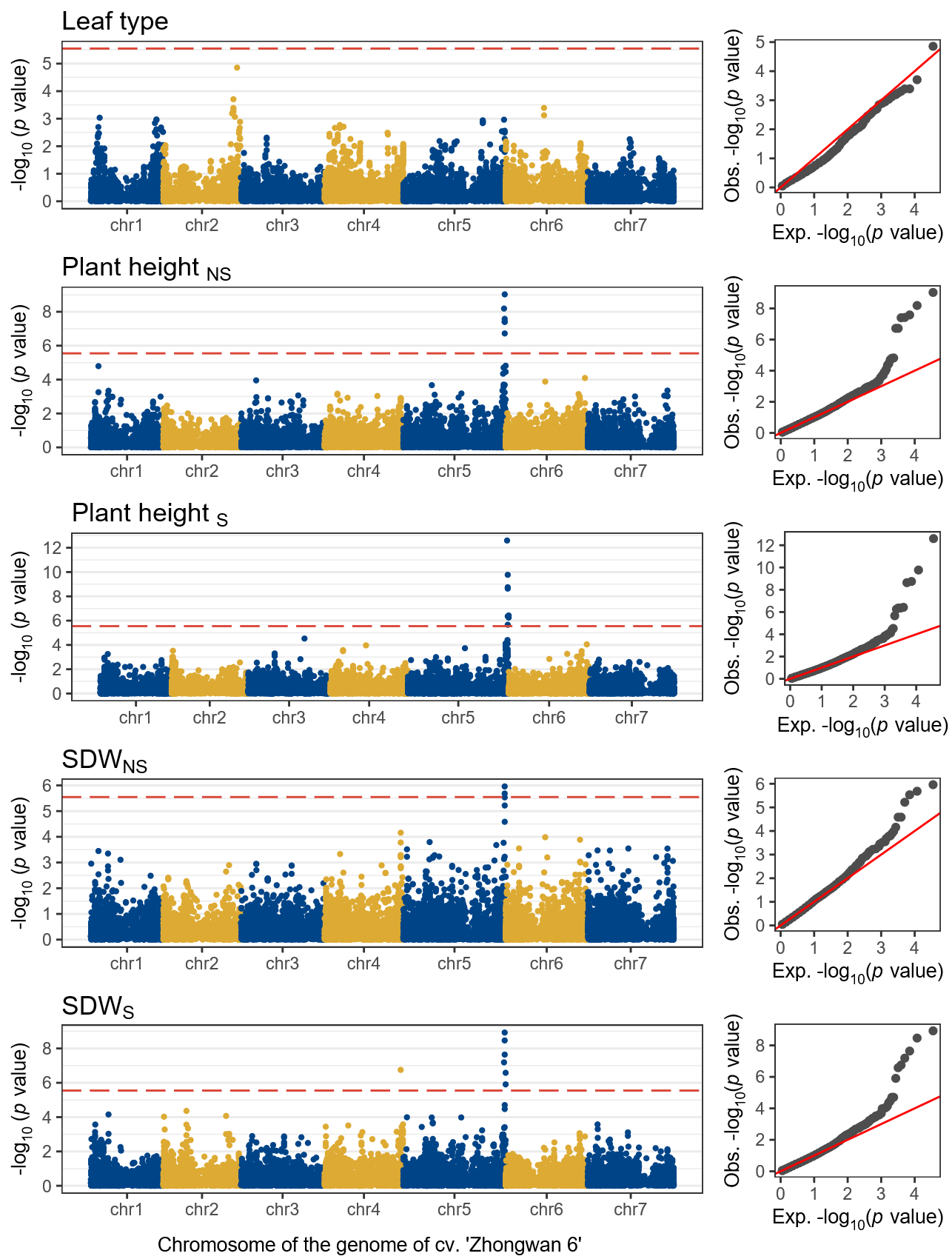


Figure S3. Results of the genome-wide association studies for the traits leaf type, plant height and shoot dry weight (SDW) under naturally infested (NS) or sterilized soil conditions (S). The horizontal, red-dashed line represents the Bonferroni-corrected threshold, which was calculated with a genome-wide type I error rate α = 0.05 (*p* < 2.028×10^-6^). The results are presented as individual Manhattan plots showing the marker-trait association significance (*y* axis) of the single nucleotide polymorphisms (SNP) and their physical location on each of the seven chromosomes of the reference genome of cv. ‘Zhongwan 6’ (*x* axis). The corresponding quantile-quantile-plots to the right compare the deviation between the observed and the expected significance of the SNP from a theoretical *Χ*^2^ distribution.


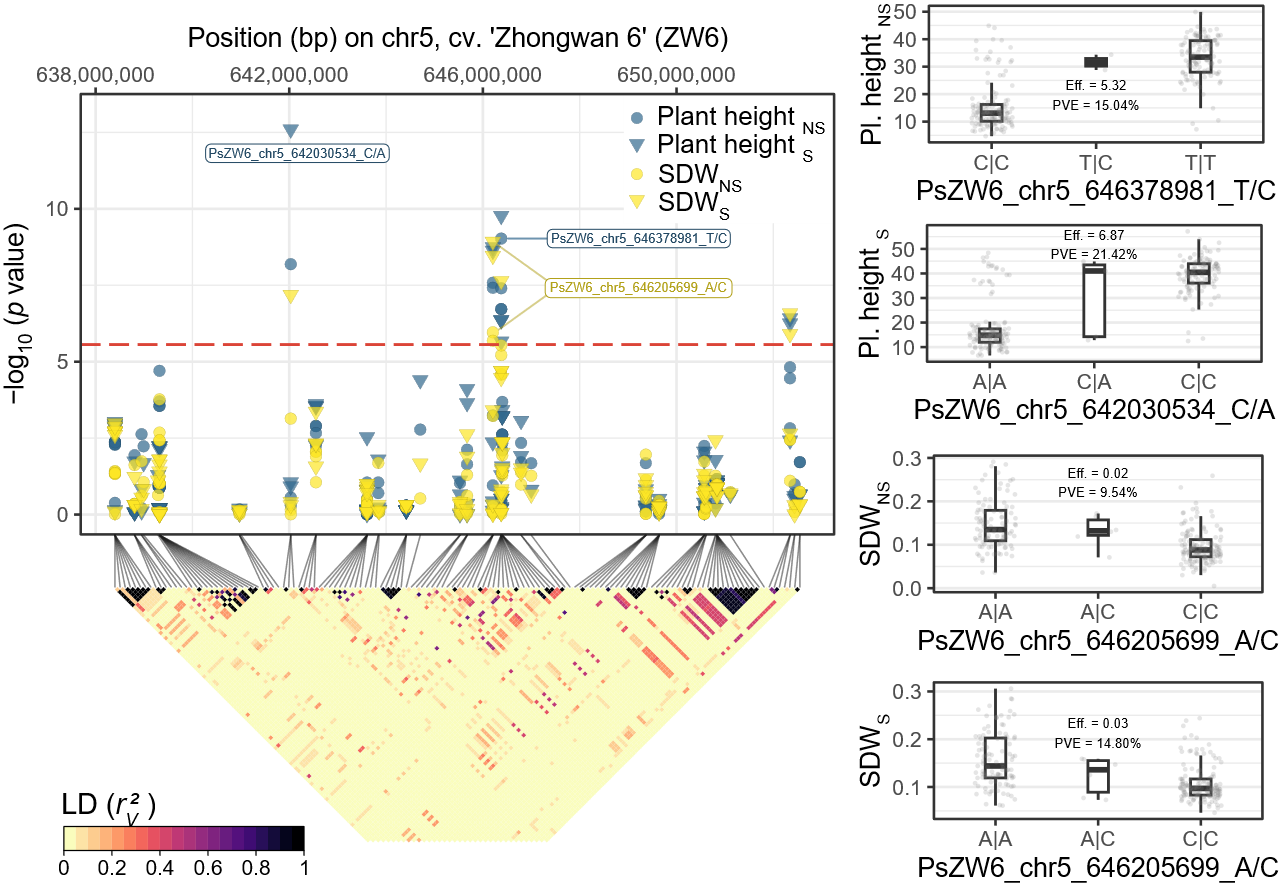


Figure S4. Genetic dissection of the region of association on chromosome chr5 for the traits plant height and shoot dry weight (SDW) under naturally infested (NS) and sterilized (S) soil conditions. The scatterplot shows combined results of individual genome wide association studies (GWAS). Each point shows the marker-trait association significance (*y* axis) of each single nucleotide polymorphism (SNP) and their physical location on chr5 of the genome of cv. ‘Zhongwan 6’ (*x* axis). The horizontal, red-dashed line represents the Bonferroni-corrected threshold, which was calculated with a genome-wide type I error rate α = 0.05 (*p* < 2.028×10^-6^). The tagged points indicate the most significant SNPs for each trait. The colored square matrix below represents the pairwise linkage disequilibrium (LD) measurements (*r_v_*²) between each pair of SNPs in the region of association. The boxplots show the distribution of phenotypic values between the genotypes of the most significant SNPs on chr5. Each plot includes the marker effect (Eff.) and the proportion of variance explained (PVE) derived from the GWAS model.


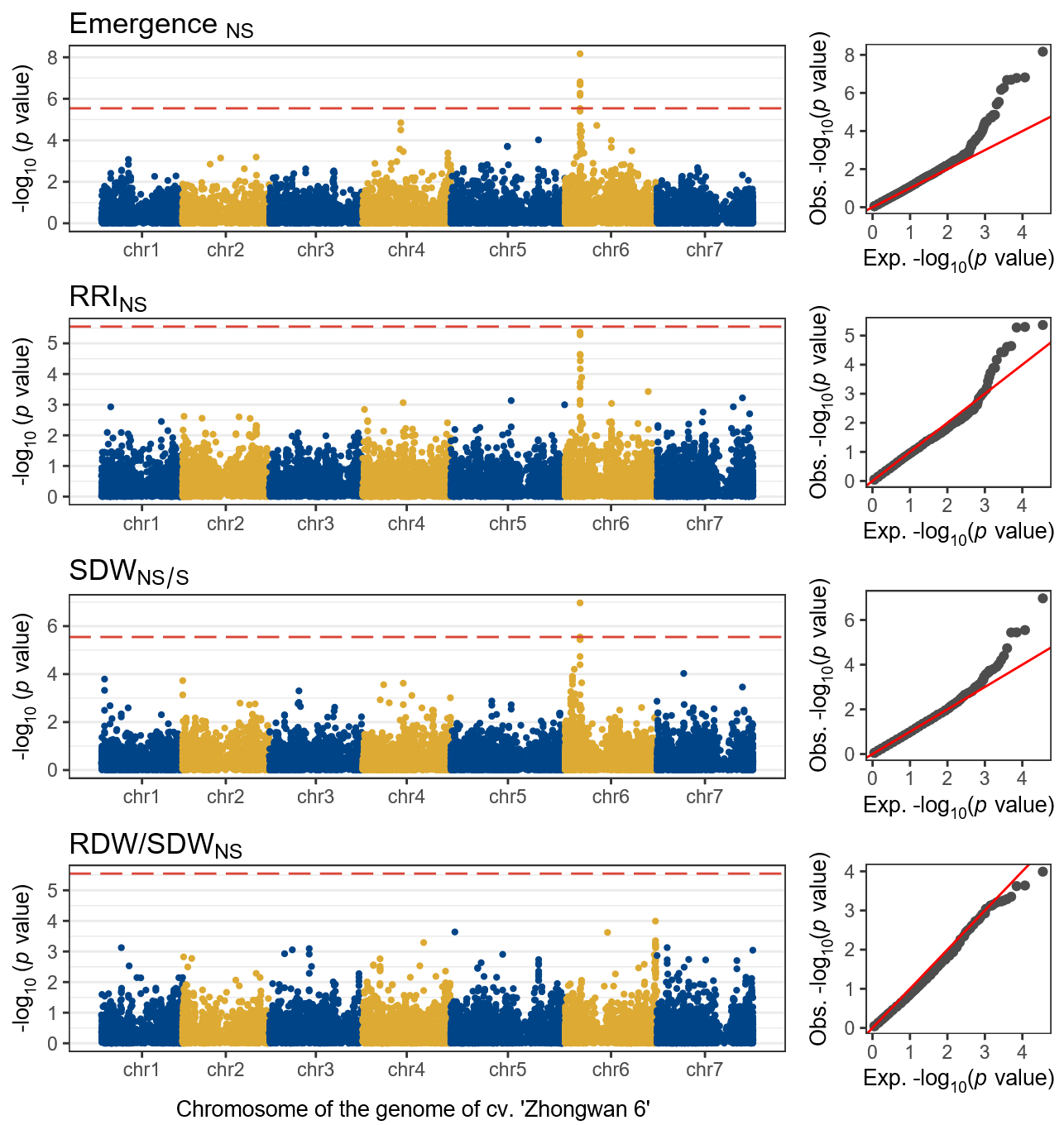


Figure S5. Results of the genome-wide association studies for the root rot-related traits plant emergence and root rot index (RRI) under naturally infested soil conditions (NS), shoot dry weight (SDW) ratio between naturally infested and sterilized soil conditions (NS/S), and the ratio between root and shoot dry weight (RDW/SDW) under NS conditions. The horizontal, red-dashed line represents the Bonferroni-corrected threshold, which was calculated with a genome-wide type I error rate α = 0.05 (*p* < 2.028×10^-6^). The results are presented as individual Manhattan plots showing the marker-trait association significance (*y* axis) of the single nucleotide polymorphism (SNP) and their physical location on each of the seven chromosomes of the reference genome of cv. ‘Zhongwan 6’ (*x* axis). The corresponding quantile-quantile-plots to the right compare the deviation between the observed and the expected significance of the SNP from a theoretical *Χ*^2^ distribution.


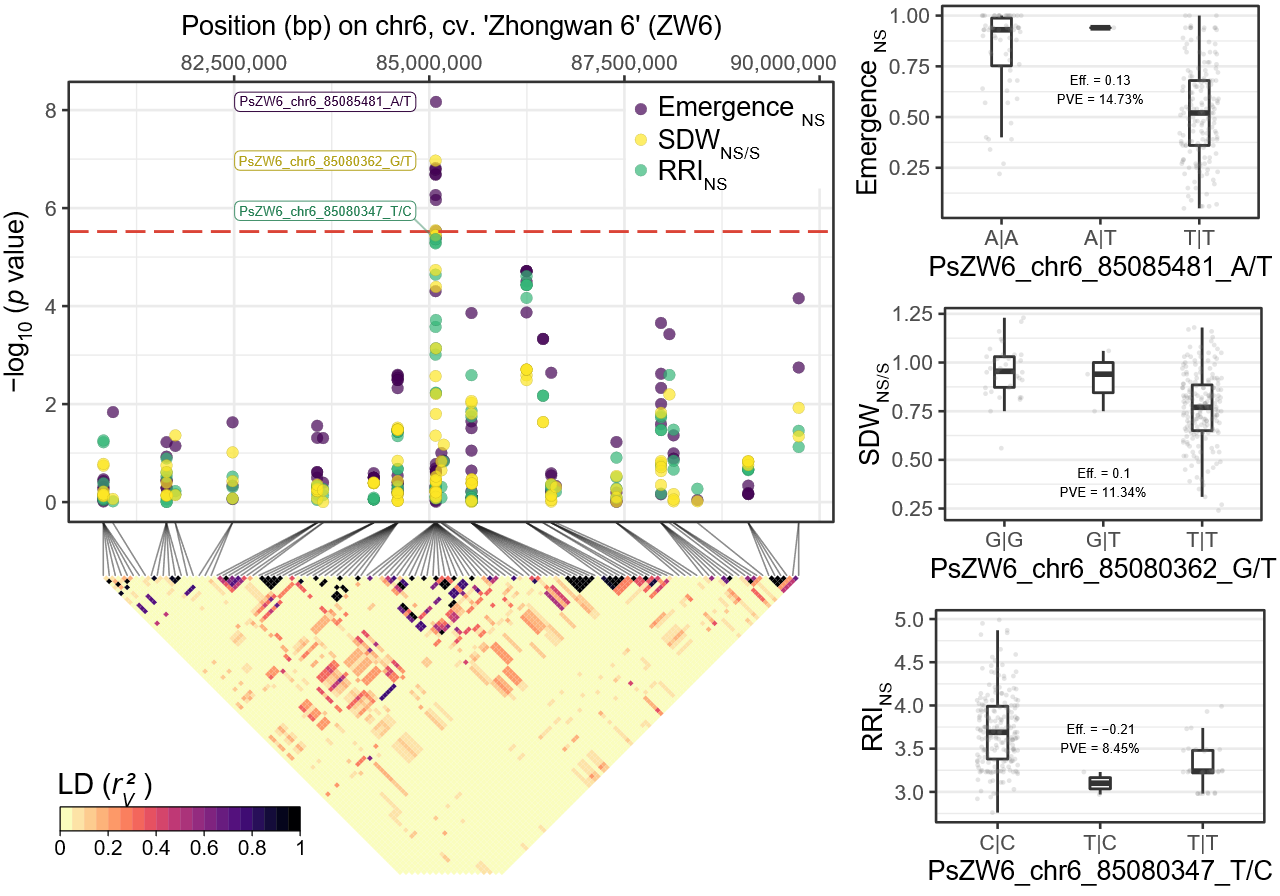


Figure S6. Genetic dissection of the region of association on chromosome chr6 for the root rot-related traits plant emergence, root rot index and shoot dry weight (SDW) under naturally infested (NS) and sterilized (S) soil conditions. The scatterplot shows combined results of individual genome wide association studies (GWAS). Each point shows the marker-trait association significance (*y* axis) of each single nucleotide polymorphism (SNP) and their physical location on chr6 in the genome of cv. ‘Zhongwan 6’ (*x* axis). The horizontal, red-dashed line represents the Bonferroni-corrected threshold, which was calculated with a genome-wide type I error rate α = 0.05 (*p* < 2.028×10^-6^). The tagged points indicate the most significant SNPs for each trait. The colored square matrix below represents the pairwise linkage disequilibrium (LD) measurements (*r_v_*²) between each pair of SNPs in the region of association. The boxplots show the distribution of phenotypic values between the genotypes of the most significant SNPs on chr6. Each plot includes the marker effect (Eff.) and the proportion of variance explained (PVE) derived from the GWAS model.


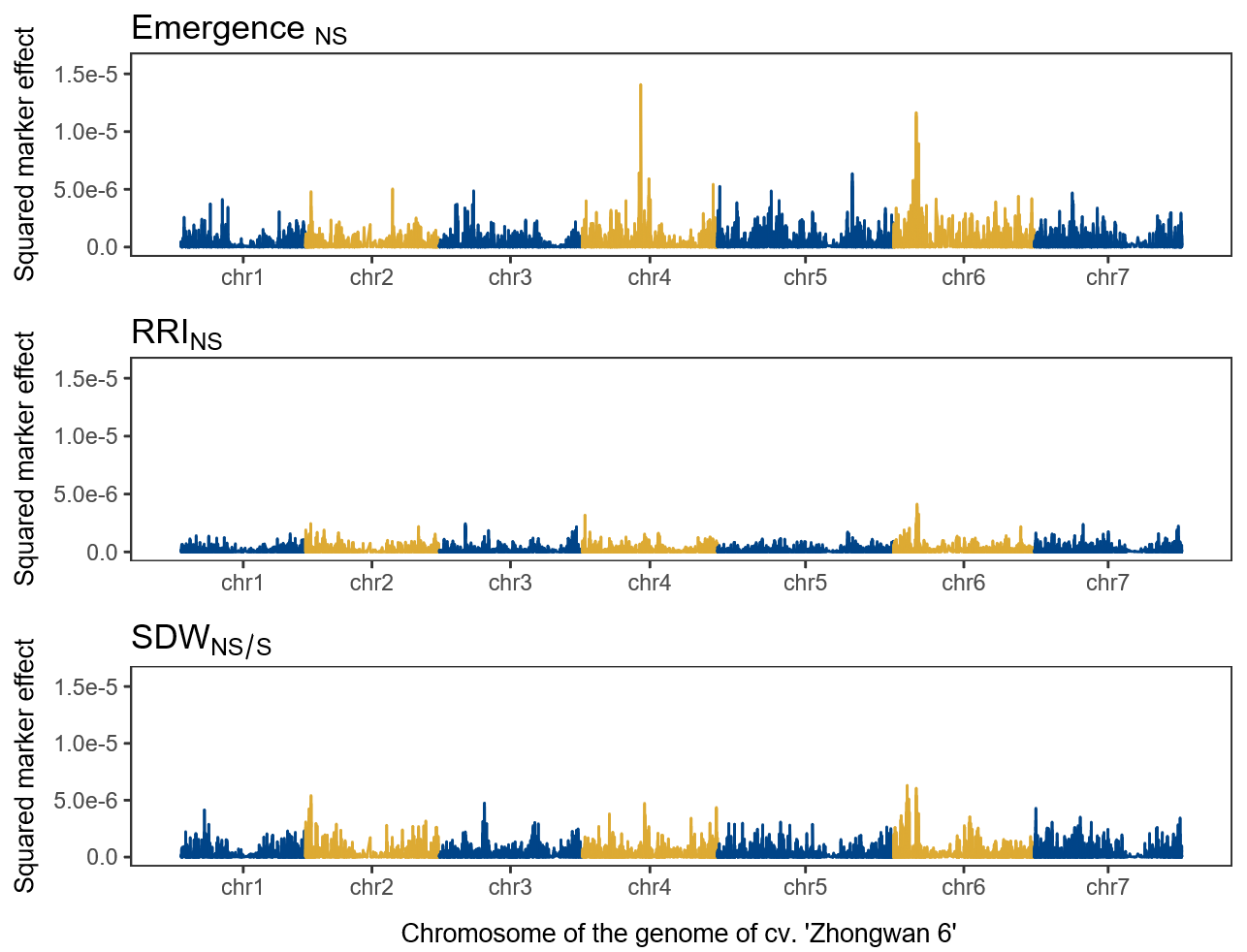


Figure S7. Single nucleotide polymorphism (SNP) effects derived from a Bayes ridge regression model that used all SNP markers as predictors of root rot-related traits plant emergence and root rot index (RRI) under naturally infested soil conditions (NS), shoot dry weight (SDW) ratio between naturally infested and sterilized soil conditions (NS/S). The squared marker effects (*y* axis) are plotted along their physical position on each one of the seven chromosomes of the reference genome ‘Zhongwan 6’ (*x* axis).


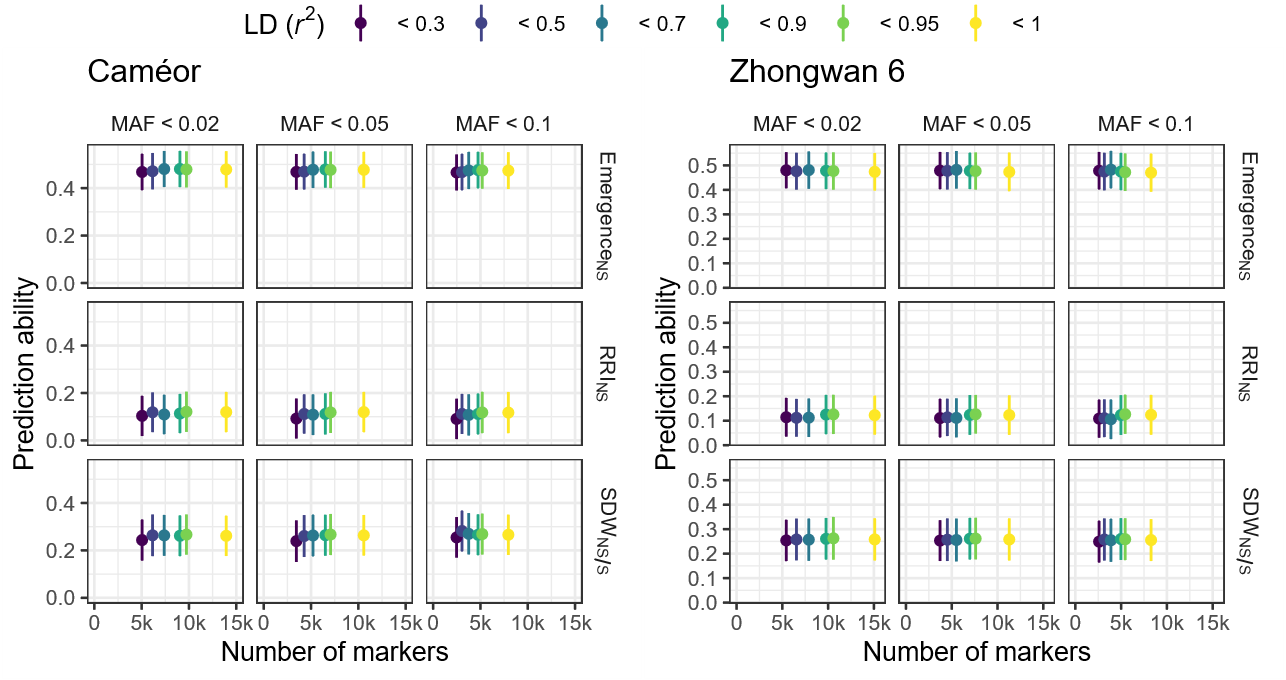


Figure S8. Prediction ability under varying marker density and minor allele frequency (MAF) thresholds. Marker density was adjusted through linkage disequilibrium (LD) pruning, retaining markers with pairwise genotypic correlation (*r*^2^) below specified thresholds within sliding windows per chromosome. Predictions were made for root rot-related traits: shoot dry weight (SDW), plant emergence, and root rot index (RRI) under naturally infested (NS), sterilized soil (S), or as NS/S ratios. Analyses were performed using the reference genomes of cultivars ‘Caméor’ and ‘Zhongwan 6’. Points represent mean prediction ability, and error bars indicate standard deviation, calculated from Pearson’s correlation between observed and predicted values in a validation subset (30%) across 50 cross-validation replicates.

Table S1. List of plant material and phenotypic data used in this study, originally reported by Wille et al. (2020). Raw genotypic data for each genotype were deposited in the NCBI Sequence Read Archive (SRA) under the corresponding BioSample and SRA run identifiers. The traits plant height, shoot and root dry weight (SDW and RDW), plant emergence and root rot index (RRI) were evaluated under naturally infested (NS) or sterilized (S) soil conditions, where NS/S indicates the ratio between them (Excel file).
